# Academic stress and mental fatigue predict subjective but not objective internal load in adolescent soccer players—a prospective cohort study

**DOI:** 10.3389/fpsyg.2026.1770781

**Published:** 2026-03-17

**Authors:** Rick Nijland, Tynke Toering, Andreas Ivarsson, Johan de Jong, Koen A. P. M. Lemmink

**Affiliations:** 1 Centre of Expertise Healthy Ageing, Sports Science, Healthy Lifestyle, Sports and Physical Activity, Hanze University of Applied Sciences, Groningen, Netherlands; 2 Department of Human Movement Sciences, University of Groningen, University Medical Center Groningen, Groningen, Netherlands; 3 School of Health and Welfare, Halmstad University, Halmstad, Sweden; 4 Department of Sport Science and Physical Education, University of Agder, Kristiansand, Norway

**Keywords:** academic stress, adolescents, mental fatigue, non-training stress, soccer, training load

## Abstract

**Introduction:**

Adolescent soccer players aspiring to reach the elite level are usually involved in a dual career. Moreover, they experience rapid physical and psychological development with unique social environments. Non-training stressors, such as educational responsibilities, can induce stress and mental fatigue, potentially negatively affecting training load. The relationship between non-training stressors and training load has received little investigation. Therefore, this study aimed to investigate the relationship between academic stress, mental fatigue and training load; specifically, the extent to which academic stress and mental fatigue predict internal load.

**Methods:**

Using a prospective cohort study design, 35 players from an Under-16 and Under-18 youth academy team were monitored daily for half a season. Measures for mental fatigue, academic stress, external load and internal load were collected before, during or after on-field training sessions. Using multilevel modelling, academic stress, mental fatigue and external load indicators served as independent variables to predict the rating of perceived exertion and the heart-rate-derived training impulse (TRIMP).

**Results:**

Final multilevel models indicated that academic stress and mental fatigue were positively associated with rating of perceived exertion, but academic stress and mental fatigue did not predict TRIMP after controlling for external load indicators.

**Conclusion:**

We conclude that academic stress and mental fatigue could lead to unwanted alterations in the rating of perceived exertion. Coaches should therefore remain alert to deviations from normal scores and relatively high levels of academic stress or mental fatigue. Given the multifaceted nature of non-training stressors, a holistic monitoring approach is recommended.

## 1 Introduction

Adolescent academy soccer players train multiple times a week and regularly play competitive matches, intending to reach the professional level. Staff at professional youth academies train and guide these talented players to create an optimal learning environment. At this level, physical prowess has become more important in recent years as physical demands during match-play have gradually increased (Allen et al., 2024). Therefore, optimal training load management is imperative to aid physical development (Murray, 2017).

Training load is a higher-order construct consisting of external load and internal load (Impellizzeri et al., 2022; Jeffries et al., 2022). External load is everything an athlete does and can be measured (Jeffries et al., 2022). Internal load is the corresponding psychophysiological response and is considered the main stimulus for training adaptations (Jeffries et al., 2022; Impellizzeri et al., 2019). Hence, practitioners should strive for the ideal internal load for each training session through training prescription. When done optimally, this leads to positive training effects whilst limiting the negative effects, which consequently should lead to improved sports performance (Jeffries et al., 2022). Nevertheless, factors from outside the physical training process, such as non-training stressors, could impact training load (Jeffries et al., 2022). The potential effect of non-training stressors on training load might have different ramifications for adolescent players than adults. Firstly, they experience rapid physical and psychological development and have a unique social environment compared to adults (Murray, 2017; Salmela-Aro, 2011; Soligard et al., 2016). Secondly, long-term development is important for academy players, while immediate performance is essential for adults (Murray, 2017). Therefore, a holistic approach to training load monitoring is recommended, specifically for adolescent players (Murray, 2017).

Non-training stressors can be described as factors or demands that cause stress, are unrelated to the physical training process, but potentially do affect it (Ivarsson and Johnson, 2010; Mellalieu et al., 2021; Jeffries et al., 2022). Although no uniform definition of stress exists, common descriptions refer to stress as a psychophysiological state that involves negative reactions and feelings which occur when external demands exceed a player’s ability to cope with these demands (Gomes et al., 2022; Lopes Dos Santos et al., 2020; Mellalieu et al., 2021). The latter could involve, among others, an increased heart rate, anxiety, difficulty concentrating, increased muscle tension and decreased coordination (Andersen and Williams, 1988; Gomes et al., 2022; McEwen, 2007). Usually, stress is not a single event but an ongoing transactional process between a player and their environment, where players appraise the situation and decide how to cope (Lazarus and Folkman, 1984; Mellalieu et al., 2021). Thus, stress is not a sole property of the environment or some fixed psychophysiological response within a player, but the result of a player’s perception of the current demands combined with their current resources to cope (Lazarus and Folkman, 1984). Moreover, the appraisal of the situation is seen as a core component of the stress process. An appraisal is the individual’s perception of the extent to which a situation is perceived as stressful (Mellalieu et al., 2021; Lazarus and Folkman, 1984). Thus, the subjective perception of an external demand provokes a unique response in each player (Mellalieu et al., 2021; Andersen and Williams, 1988).

Stress, including non-training stress, has been found to affect several components of the physical training process. For example, increased feelings of stress have been associated with increased risk of injury and illness (Hamlin et al., 2019; Soligard et al., 2016). Suggestions have been put forward that internal load could also be influenced by stress (Coyne et al., 2022). Mixed results, however, were found for the association between stress and internal load indicators. Two studies found a positive association between stress and Rating of Perceived Exertion (RPE), whereas another did not (Haddad et al., 2013; Long et al., 2024; Nobari et al., 2021). These results indicate that more research remains warranted.

Mental fatigue is a psychobiological state that occurs after prolonged periods of cognitive activity, combining perceptual, behavioural and physiological manifestations such as feelings of tiredness and reduced motivation (Russell et al., 2019; Van Cutsem et al., 2017; González-Víllora et al., 2022). Although the relationship between non-training stress and mental fatigue is not yet fully understood, mental fatigue and stress are considered separate constructs with unique manifestations and underlying mechanisms (Kocalevent et al., 2011; McEwen, 2007). Yet, it is thought that non-training stressors could also induce mental fatigue (Russell et al., 2019; Roelands et al., 2022; Thompson et al., 2020), making it a relevant concept to consider in this study. Moreover, research links prolonged stress with increased mental fatigue (Zhang et al., 2021; Kocalevent et al., 2011). Mental fatigue potentially affects training load, as indicated by several studies (González-Víllora et al., 2022; Clemente et al., 2021; Van Cutsem et al., 2017). However, contrasting results have also been found, particularly regarding external load (Coutinho et al., 2017; Thompson et al., 2019; Thompson et al., 2020; Badin et al., 2016). Nonetheless, it has consistently been observed that subjective measures, such as RPE, were higher during exercise when mentally fatigued (Van Cutsem et al., 2017). However, since previous studies applied specific protocols (e.g., Stroop test) to induce mental fatigue, issues were raised regarding the ecological validity of the results, questioning whether real-life causations of mental fatigue would have a similar impact (Thompson et al., 2019). Later research indicated that players experienced increased feelings of mental fatigue due to specific circumstances, such as post-match or after extensive travelling (Abbott et al., 2020; Thompson et al., 2020). Overall, given that non-training stressors are a potential cause for mental fatigue, it is reasonable to include mental fatigue when examining the relationship between non-training stressors and training load.

Non-training stressors can originate from various sources, such as organisational, performance and personal demands (Mellalieu et al., 2009; Mellalieu et al., 2021). Organisational demands are related to the organisational aspects of sports, such as wages, leadership and logistics. Performance demands are related to competition or the preparation to compete, including injuries and match performance. Finally, personal demands are associated with life events away from the sport, such as educational responsibilities and family. Many distinct demands related to organisational and performance have been identified, but personal demands have not received the same level of scrutiny in scientific literature (Fletcher et al., 2008; Mellalieu et al., 2009; Mellalieu et al., 2021). Moreover, the relationship between non-training stressors and training load remains understudied, specifically those involving personal demands (Nijland et al., 2024). Therefore, this study will focus on non-training stressors related to personal demands.

Educational responsibilities are an important personal demand for adolescent players and could act as a non-training stressor for academic stress and mental fatigue (Lopes Dos Santos et al., 2020; Hamlin et al., 2019; Henriksen et al., 2010; Thompson et al., 2020; Russell et al., 2019). Most adolescent players are involved in mandatory secondary or sometimes tertiary education. This means that academy players must balance their responsibilities in soccer and education. However, education does not have the same importance for all players; some might view education as a contingency plan in case they cannot make it into professional soccer, while others focus mainly or solely on soccer (Lopes Dos Santos et al., 2020). Regardless, balancing both responsibilities might have disadvantages. For example, the combined responsibilities could lead to feelings of stress due to time management demands (Lopes Dos Santos et al., 2020). It has also been observed that during periods of high academic stress (e.g., exam weeks), athletes tend to get ill and injured more often (Hamlin et al., 2019; Mann et al., 2016). Therefore, it has been suggested that coaches, specifically in demanding periods, take educational demands into account when planning training sessions, since these could cause stress (Hamlin et al., 2019; Mann et al., 2016).

**Figure 1:**
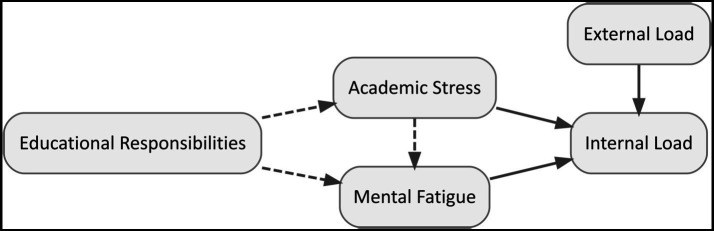
Theoretical model of relationships among educational responsibilities, academic stress, mental fatigue, and training load. Solid arrows represent associations of interest, where dashed arrows represent theoretically possible associations. Arrows do not represent causal relationships.

This study aims to gain insight into the relationship between academic stress, mental fatigue and training load in adolescent soccer players (see Figure 1). Since internal load is the main stimulus for training adaptation, the research question for this study is: To what extent do academic stress and mental fatigue predict internal load in adolescent soccer players after controlling for external load? It was hypothesised that higher academic stress and mental fatigue values are associated with higher internal load values.

## 2 Materials and methods

### 2.1 Participants

A total of 35 players (age 16.1 ± 1.2 years; length 176.0 ± 8.8 cm; body mass 64.4 ± 9.9 kg) belonging to an Under-16 (*n* = 16) and Under-18 (*n* = 19) team from a professional soccer club consented to participate in this study. For players younger than 16 years old, their parents or caregivers also provided consent. All players and parents received written information regarding the aims of the study before its commencement. Goalkeepers were excluded due to different movement patterns and because monitoring external load was deemed impractical for goalkeepers. Players from both teams trained 4 to 5 times a week, and each session lasted on average between 60 and 90 min. Furthermore, both teams played a competitive match on Saturday. In addition to the on-field sessions, players had 2 to 3 strength training sessions per week, and in some weeks, friendly matches were held on Tuesdays.

Most players were in high school (secondary education in the Netherlands), which is mandatory. A small group of players (*n* = 7) were involved in vocational education (tertiary level). Depending on the level of education, players had multiple weeks in which examinations took place during the study period. Moreover, players were required to submit several assignments throughout the academic year, based on the courses they took. Players in their senior year of high school had final exams scheduled for 2 weeks in May and possible resits in June.

### 2.2 Study design

Using a prospective observational design, players from an Under-16 and Under-18 team were monitored for 20 weeks from January to June 2023. Per team, 60 to 70 training sessions took place. Players were only monitored during regular field-based training sessions, meaning that strength and general fitness sessions at the gym, friendlies and matches were excluded due to practical reasons. During the monitoring period, measures for external load, internal load, academic stress and mental fatigue were collected daily. Before the first session of the day, players provided information through self-report measures regarding academic stress and mental fatigue, which were administered by and only accessible to the researcher team. External load was monitored using GPS trackers and accelerometers (Polar Team Pro, Polar, Helsinki, Finland) and Heart Rate (HR) for internal load. After each session, players provided RPE on a 6–20 scale (CR-20). Coaches and players were familiarised with all measurements before commencement through an interactive meeting and a two-week familiarisation period. This study was approved by the Ethics Committee of the Hanze University of Applied Sciences and conformed to the recommendations of the Declaration of Helsinki.

### 2.3 Measurement instruments

#### 2.3.1 Academic stress

We opted to measure players’ perceived academic stress daily. Therefore, a bespoke single-item question adapted from Hamlin et al. (2019) was used since multi-item questionnaires were deemed impractical and too burdensome for players to implement daily (Hamlin et al., 2019). Before commencement of the study, the question used by Hamlin et al. (2019) was translated into Dutch using back-translation procedures. First, the lead author translated the question into Dutch; thereafter, two bilingual individuals both translated it back into English without the help of the original scale. The original version and back-translated versions were then compared, and discrepancies were discussed, after which agreement was reached. Moreover, the lead author met with the head coach of each team (*n* = 2) and a small group of players (*n* = 4) to discuss the suitability of the question. It was decided to alter the word ‘pressure’ into ‘stress’ to better reflect the aim of the study; it was deemed more fitting in the native language of the players, and players indicated it better reflected how they understood the construct. Before the first training session of the day, players were asked to provide their current perceived academic stress on a 5-point scale (1 = no academic stress, 2 = normal academic day, 3 = heavy academic day, 4 = academic stress building, 5 = academic stress high). Academic stress was collected using Qualtrics XM (Provo, Utah, United States of America).

#### 2.3.2 Mental fatigue

Mental fatigue was assessed using a single-item question: “How mentally fatigued do you currently feel?” (Russell et al., 2022a; Russell et al., 2022b). To limit potential assessment bias, w provided a clear and uniform operational definition with examples of mental fatigue (Abbott et al., 2020; Thompson et al., 2020). The operational definition used by Marcora et al. (2009) was provided: “a reduced ability to perform cognitive and behavioural tasks with feelings of lethargy”(Marcora et al., 2009; Abbott et al., 2020). Furthermore, sport-specific manifestations of mental fatigue were provided, similar to those suggested by Russell et al. (2019). These manifestations included feelings of disengagement, decreased motivation and enthusiasm, increased displays of emotion and withdrawal, changes in concentration, and decreased discipline and attention to detail. Mental fatigue was measured using a digitally administered visual analogue scale with word anchors on both ends (none to maximal). Scores ranged from 0 to 100, and players were not able to view their score while completing the scale. In recent years, digital administration of visual analogue scales has become more commonplace, with research supporting comparability with paper-based visual analogue scales irrespective of the scale’s length (Byrom et al., 2022). Mental fatigue was also collected before training using Qualtrics XM.

#### 2.3.3 External load

External load was measured using Polar Team Pro with a 10 Hz GPS and 200 Hz MEMS movement sensor, which is deemed a valid measurement system (Scott et al., 2016; Sandmæl et al., 2023). From the GPS-sensor, the following external load indicators were included: total distance in meters (TD) and distance covered in meters at ≥ 19.8 m/s (High-speed running; HSR). Moreover, the number of accelerations over 2 m/s^2^ (ACC) and the number of decelerations under −2 m/s^2^ (DEC) were measured using the 200 Hz MEMS sensor. These external load indicators were identified in earlier studies as important predictors for internal load (Coutinho et al., 2015; Jaspers et al., 2018; McLaren et al., 2018).

#### 2.3.4 Internal load

##### 2.3.4.1 Rating of perceived exertion

Internal load was measured using a subjective self-report measure for RPE, namely the original Borg scale (CR-20). The CR-20 measures perceived exertion on a scale ranging from 6 (no exertion) to 20 (maximal exertion). The CR-20 is a commonly used and valid method for measuring internal load in soccer players (Impellizzeri et al., 2004). Approximately 30 min after a training session, players provided their CR-20 score for the whole training session, starting from the warming-up until cooling-down, including the time players stood still for instructions. RPE was collected using the club’s bespoke athlete monitoring system, and the CR-20 was already part of the club’s standard monitoring practice. Thus, players already had ample experience with the scale administration before the start of the current project.

##### 2.3.4.2 Training impulse

Internal load was also measured using HR. HR was recorded using Polar Team Pro with a 1-Hz frequency. The Training Impulse (TRIMP) was calculated using HR according to the formula of Stagno to quantify IL based on HR (Stagno et al., 2007). Players’ HR was stratified into five zones based on percentages of their individual maximal HR, with the lowest zone starting at 65% of HRmax. The total time (min.) spent in each zone was calculated and multiplied by a zone-specific weighting factor. Weighted zone scores were then summed to derive the training impulse (TRIMP) score.

### 2.4 Statistical analysis

All raw data were exported from their respective applications to CSV or Microsoft Excel files, which were then processed using R and Rstudio (version 2025.05.1 + 513). Raw data files from Polar Team Pro were imported into R, and external load and internal load indicators were calculated for each training session per player. Raw data from questionnaires (RPE, mental fatigue, and academic stress) were imported into R and combined with the Polar Team Pro data and player information (e.g., pseudonym and team). Mean and standard deviation were calculated for all variables separately for the Under-16, Under-18, and the combined group. Differences between the two teams in questionnaire scores, external load, and internal load indicators were assessed using multilevel models, accounting for repeated measures within players. Estimated marginal means and pairwise comparisons were used to obtain adjusted group means and associated *p*-values.

Several checks were performed to ensure the quality of the data. This includes removing non-sensical values, files with technical issues (e.g., 21 for RPE or substantial missing GPS data) and removing non-training data. Training sessions lasting less than 30 min were removed because the coaches indicated these were not regular training sessions.

Missing data were present in the dataset. Of all measured constructs (e.g., excluding pseudonym, date), ~31% of the data was missing. Missing data pattern analysis likely indicated the data were missing at random instead of missing completely at random or missing not at random. Moreover, Little’s missing completely at random test was significant (see supplemental material in the repository). Therefore, we reasonably assumed the data to be missing at random.

To handle missing data, we used multiple imputation as listwise deletion is not recommended, as this likely creates biased results and reduces power (Woods et al., 2024; van Buuren, 2018). However, to explore existing relationships within the data, we first build multilevel models for RPE and TRIMP with the existing dataset (see supplemental material in the repository). First, the null-model was specified, after which the multilevel model was gradually expanded with level-1 predictors, level-2 predictors, tested for random slopes, and finally, interaction effects were investigated. Random slopes and interactions did not improve the model. Thereafter, we used the information about the relationships to create the imputation model since the imputation and analytical models need to be congenial (van Buuren, 2018; Woods et al., 2024; Woods et al., 2021). Moreover, the imputation model is more general to improve the imputation process (van Buuren, 2018). The ‘mice’ package (van Buuren and Groothuis-Oudshoorn, 2011) was utilized for multiple imputation, along with the ‘miceadds’, ‘mitml’, and ‘broom.mixed’ packages for further analyses and model diagnostics. Before multiple imputation, 1 player was removed due to missing more than 90% of the responses on academic stress and mental fatigue. Fully conditional specification with Markov Chain Monte Carlo equations was used to set up the imputation model. Training sessions (level 1) were clustered in players (level 2). For the level-1 variables, 2-level predictive mean matching was used to impute the variables (van Buuren, 2018; Bache-Mathiesen et al., 2022). In the imputation model, auxiliary variables were included that aided in prediction or were related to missingness (van Buuren, 2018; Woods et al., 2021). These auxiliary variables included the cluster means of several variables (academic stress, mental fatigue, TD, HSR, ACC, DEC, TRIMP and RPE), training duration, weekday, week number, training type (e.g., Matchday-1, Matchday-2, Matchday+2) and players’ playing position. In the imputation model, we included random slopes for academic stress and mental fatigue based on theoretical assumptions. The analytical model and imputation model were congenial. Some variables (e.g., those for external load) were excluded from predicting each other due to computational issues (van Buuren, 2018). In total, we produced *m =* 31 multiple imputed datasets following advice to set the number of *m* to the percentage of missing data (van Buuren, 2018). Model diagnostics showed that the multiple imputation process converged, and the completed datasets were deemed acceptable.

After multiple imputation, we used multilevel modelling to predict RPE and TRIMP scores with 2 separate models. Since training sessions were nested within players, a multilevel model is appropriate to deal with the data structure. The level-1 variables consisted of RPE, TRIMP, TD, HSR, ACC, DEC, mental fatigue and academic stress. We included ‘Team’ as a level-2 variable. We checked for random slopes for academic stress and mental fatigue using a D4 test using the ‘mitml’ package, because from a theoretical stance, random slopes might be expected. Categorical variables were inserted into the model with effects coding. Final models converged and were estimated using Restricted Maximum Likelihood. Results from the 31 completed datasets were pooled using Rubin’s Rules.

To assess the robustness of our findings, we conducted several sensitivity analyses to investigate different underlying assumptions of multiple imputation. The sensitivity analyses included using ‘2 L.norm’ as a predictor method, removing random slopes for academic stress and mental fatigue, and delta-simulations (e.g., adding 1 or 10 to RPE, academic stress and mental fatigue). Also, identical analyses were conducted using the complete-case dataset.

## 3 Results

### 3.1 Descriptive statistics

Table 1 contains the general descriptives for the external load indicators, internal load indicators, academic stress and mental fatigue. In general, Under-16 players trained slightly longer and demonstrated marginally higher values for most external load and all internal load indicators. HSR and ACC were the only external load indicators that did not significantly differ between the teams. Moreover, values for both academic stress and mental fatigue did not differ significantly between the Under-16 and Under-18.

**Table 1:** General descriptives for external load indicators, Internal load indicators, perceived academic stress and mental fatigue for the total group, under-16 and under-18.

Measure category	Overall (*N* = 35)	Under-16 (*N* = 16)	Under-18 (*N* = 19)
External Load indicators			
Duration (min)	74.2 ± 15.7	76.1 ± 15.4	72.7 ± 15.9†
Total distance (m)	4942.3 ± 1244.0	5105.7 ± 1174.6	4808.3 ± 1283.5†
LSA (m)	4189.9 ± 966.7	4274.4 ± 843.5	4120.6 ± 1052.7‡
MSR (m)	562.9 ± 271.3	626.2 ± 310.1	511.0 ± 222.0†
HSR (m)	189.5 ± 165.6	205.1 ± 190.0	176.7 ± 141.3
Accelerations > 2 m.s^−2^ (no)	71.3 ± 21.9	72.0 ± 23.3	70.7 ± 20.8
Decelerations < −2 m.s^−2^ (no)	70.3 ± 22.5	73.3 ± 24.7	67.9 ± 20.3*
Internal load indicators			
RPE (AU)	13.3 ± 2.3	14.1 ± 2.5	12.9 ± 2.1†
TRIMP (AU)	129.4 ± 47.6	143.5 ± 44.2	117.8 ± 47.1†
Questionnaires			
Academic stress (AU)	2.0 ± 0.7	2.1 ± 0.7	2.0 ± 0.6
Mental fatigue (mm)	23.6 ± 14.3	22.7 ± 14.1	24.2 ± 14.5

LSA, Low speed activities (0–14.4 km.h^−1^); MSR, Moderate Speed Running (14.4–19.8 km.h^−1^); HSR, High Speed Running (>19.8 km.h^−1^); RPE, Rating of Perceived Exertion; TRIMP, Training Impulse. † *p* < 0.001; ‡ *p* < 0.01; * *p* < 0.05.

### 3.2 Multilevel models

#### 3.2.1 RPE

Table 2 presents the results of the multilevel model predicting RPE. The intraclass correlation coefficient for this model was 0.1625, indicating that 16.25% of the variance is attributed to differences between players. The intercepts showed significant variance across players (b = 14.143, SE = 0.580, t(460.719) = 24.399, *p* < 0.001).

**Table 2:** Model parameters for the final multilevel model for predicting RPE.

Fixed effects	Estimate	S.E.	D.F.	*P*-value
Intercept	14.143	0.580	460.719	<0.001
Academic stress	0.267	0.111	159.220	0.018
Mental fatigue	0.018	0.006	88.966	0.006
Total distance	0.0003	0.0001	55.613	0.009
High-speed running	0.003	0.001	86.618	<0.001
Accelerations	0.005	0.005	116.275	0.340
Decelerations	0.002	0.005	118.056	0.655
Team	−0.870	0.317	875.238	0.006

Random effects	Variance
Intercept	0.666
Residual	3.944

S.E., Standard Error; D.F., Degrees of Freedom. For total distance, 4 decimal places are used instead of 3 to prevent masking meaningful effects.

Both academic stress (b = 0.267, t(159.220) = 2.394, *p* = 0.018) and mental fatigue (b = 0.018, t(88.996) = 2.837, *p* = 0.006) have a significant direct effect on RPE after controlling for external load indicators and team. Moreover, TD (b = 0.0003, t(55.613) = 2.710, *p* = 0.009), HSR (b = 0.003, t(86.618) = 5.139, p < 0.001) and team (b = −0.870, t(558.78) = −2.741, p = 0.006) were also significant predictors for RPE.

D4 tests comparing models with and without slopes for academic stress (*F*(2, 505.705) = 1.125, *p* = 0.326) and mental fatigue (*F*(2, 659.643) = 0.444, *p* = 0.432) indicated no significant improvement.

#### 3.2.2 Modified TRIMP

Table 3 presents the results of the multilevel model predicting TRIMP. The intraclass correlation coefficient was 0.2071, indicating that 20.71% of the variance was attributed to differences between subjects. The intercepts showed significant variance across players (b = 155.496, SE = 13.323, t(984.353) = 11.671, *p* < 0.001).

**Table 3:** Model parameters for the final multilevel model for predicting TRIMP.

Fixed effects	Estimate	Standard error	DF	*P*-value
Intercept	155.496	13.323	984.353	<0.001
Academic stress	0.555	2.335	79.823	0.813
Mental fatigue	0.077	0.104	117.604	0.459
Total distance	0.010	0.002	69.138	<0.001
High-speed running	0.018	0.009	120.434	0.040
Accelerations	0.279	0.090	103.920	0.003
Decelerations	0.437	0.095	88.755	<0.001
Team	−17.507	7.841	1226.432	0.026

Random effects	Variance
Intercept	459.944
Residual	1204.880

Academic stress (b = 0.555, t(79.823) = 0.238, *p* = 0.813) and mental fatigue (b = 0.077, t(117.604) = 0.743, *p* = 0.459) were not significant predictors for TRIMP. However, TD (b = 0.010, t(69.138) = 6.574, p < 0.001), HSR (b = 0.018, t(120.434) = 2.077, *p* = 0.040), ACC (b = 0.279, t(103.920) = 3.083, *p* = 0.003), DEC (b = 0.437, t(88.755) = 4.590, p < 0.001) and team membership (b = −17.507, t(1226.432) = −2.233, *p* = 0.026) were significant predictors for TRIMP.

D4 tests comparing models with and without slopes for academic stress (*F*(2, 319.660) = 0.121, *p* = 0.886) and mental fatigue (*F*(2, 504.458) = 0.166, *p* = 0.847) indicated no significant improvement.

#### 3.2.3 Anecdotal example

**Figure 2:**
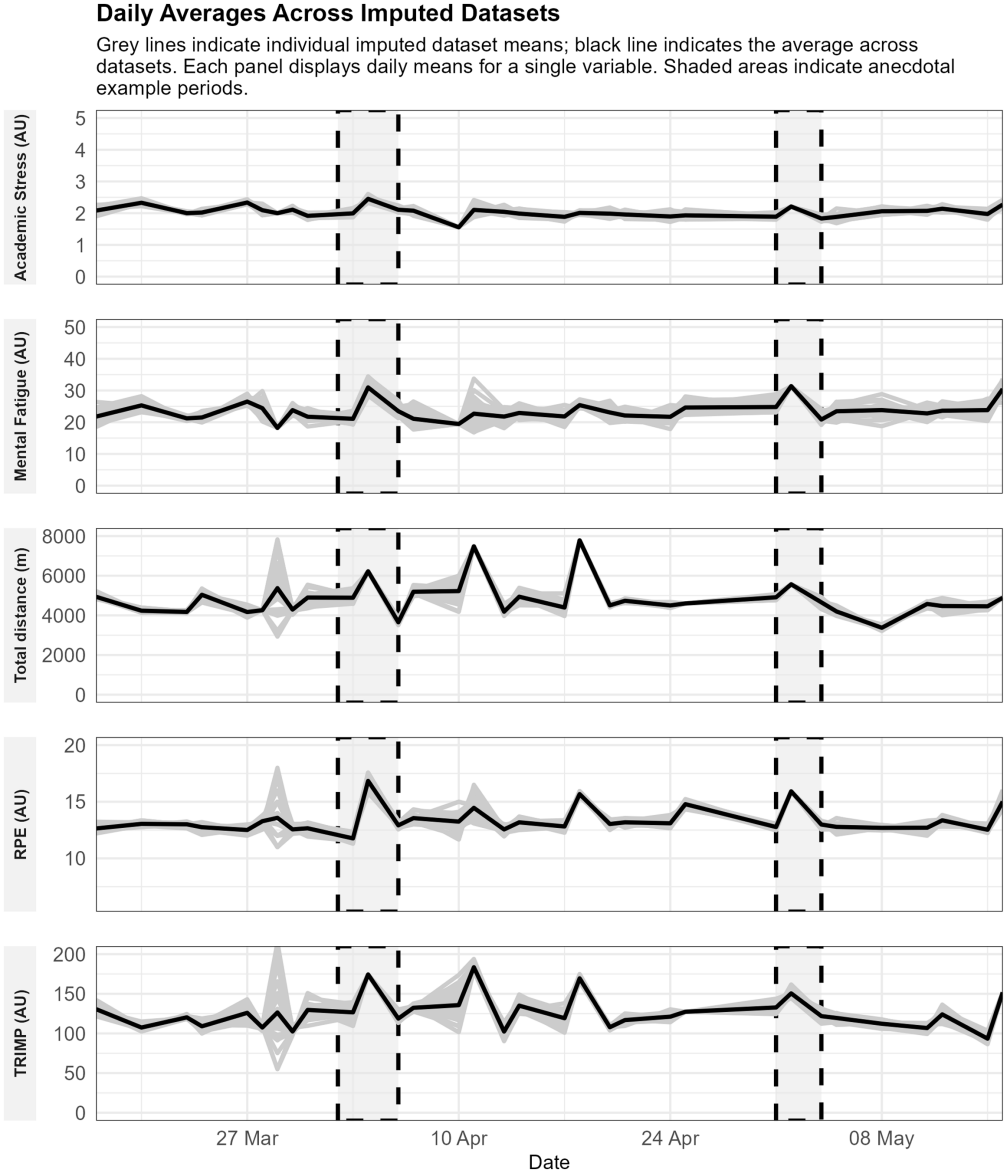
Anecdotal example of the daily averages across imputed datasets for, from top to bottom, academic stress, mental fatigue, total distance, rating of perceived exertion (RPE), and training impulse (TRIMP).

Figure 2 shows two anecdotal examples depicting a period (marked with dashed rectangles) when a subset of players had an examination week coinciding with an increase in academic stress, mental fatigue and RPE and to a lesser degree, TD and TRIMP as well. It is worth noting that these patterns were not always evident in the average scores of all players. The grey lines depict the mean daily scores of all players within each imputed dataset, calculated separately for every dataset. In contrast, the black line represents the aggregated mean across all imputed datasets.

### 3.3 Sensitivity analysis

The sensitivity analyses showed that only when academic stress was underestimated during multiple imputation did it cease to be a significant predictor of RPE (see supplementary file). Across all other sensitivity analyses, estimates for academic stress and mental fatigue remained consistent for both RPE and TRIMP, supporting the overall robustness of the main findings.

## 4 Discussion

It is common for adolescent soccer players to combine their soccer aspirations with educational responsibilities. These could act as a non-training stressor and potentially influence training load through academic stress and/or mental fatigue. To our knowledge, this is the first study to examine the relationship between academic stress, mental fatigue and training load in adolescent soccer players using a combined physiological and sociopsychological measurement approach. Our data demonstrate that academic stress and mental fatigue have varying associations with internal load. Specifically, RPE tends to increase with increasing academic stress and mental fatigue, but neither academic stress nor mental fatigue were associated with TRIMP. Furthermore, the final models did not include random slopes, indicating that the overall strength of the relationship was similar for all players. An important observation is that players did not often report academic stress and mental fatigue values in the fourth quartile. Given that such values likely have the largest consequences in practice, it seems recommendable to research such cases more in-depth, both practically and academically. Additionally, results indicate the importance of monitoring relatively big changes in academic stress and mental fatigue scores, regardless of absolute scores.

### 4.1 Discussion of results

Previously, the relationship between non-training stressors and training load has been suggested but hardly measured; to the best of our knowledge, this is the first study to investigate this relationship for academic stress using an observational design in a real-world setting (Coyne et al., 2022; Mellalieu et al., 2021). Academic stress was positively associated with RPE, corroborating the previous suggestions. Practitioners working with adolescent soccer players should be heedful of this relationship because educational responsibilities often form an important part of an adolescent’s personal life. Unwanted internal load alterations could occur, potentially leading to unexpected training outcomes. Additionally, previous research highlighted the importance of monitoring non-training stress due to its association with increased risk of illness or injury and changes in players’ well-being (Hamlin et al., 2019; Saw et al., 2016). This all points to the importance of taking into account players’ dual career pathways to optimise their performance, development and wellbeing; particularly because relatively few academy players end up making a living out of soccer (Grossmann and Lames, 2015).

Recent evidence indicated that youth academy players experience low to moderate levels of mental fatigue (Thompson et al., 2020; Abbott et al., 2020). Similar values were observed in this study, and additionally, we found that higher scores of mental fatigue also tended to lead to higher scores of RPE (Van Cutsem et al., 2017). Others found mental fatigue could be elevated based on external factors, such as match success, travel or education (Thompson et al., 2020; Abbott et al., 2020; Russell et al., 2019). Although this study did not investigate the relationship between educational responsibilities and mental fatigue, nor between academic stress and mental fatigue, it is likely that both factors contribute, at least in part, to mental fatigue levels. From a theoretical perspective, mental fatigue relates to demands in general rather than solely educational responsibilities, highlighting the need for holistic training load monitoring practices given the multifaceted nature of stressors in the training environment (Coyne et al., 2018; Nijland et al., 2024; Murray, 2017). Overall, it seems relevant to include mental fatigue in the daily monitoring practice, which allows practitioners to implement strategies to optimise daily training load management (Russell et al., 2022a).

An important observation is that players mostly reported pre-training academic stress and mental fatigue values in the first or second quartile of the scale, indicating a low to moderate presence of academic stress and mental fatigue. This is not unsurprising since training sessions were performed in the morning, and sleep, for example, is expected to counter mental fatigue (Martin et al., 2018). Interestingly, a recent study found that players who reported longer sleep duration and better sleep quality experience less mental fatigue, but this effect increases as the season progresses (Long et al., 2025). Furthermore, since multilevel analysis separates between-group from within-group variance, our results suggest that the effect of academic stress and mental fatigue on RPE is similar across players. This is further corroborated by the sensitivity analyses, which generally confirmed the robustness of the main findings. Therefore, supporting staff should be attentive when individual players deviate significantly from their normal scores, instead of looking at the absolute score only or at group averages (see also Figure 2). An interesting avenue of research could be investigating a potential temporal effect of academic stress and mental fatigue on internal load, since previous research indicates that these relationships might change as the season progresses (Long et al., 2025). Ideally, these studies should also include other factors that may influence internal load, such as sleep and other well-being measures. Nevertheless, researchers must ensure a balance between the study’s aim and the burden for players and staff, to increase staff and player buy-in and avoid questionnaire fatigue (Saw et al., 2015).

Although this study focused on academic stress, non-training stressors can stem from various sources contributing to stress or mental fatigue. Stress is an individual transactional process, with earlier experiences affecting individual responses when facing new situations (McEwen, 2007; Lazarus and Folkman, 1984; Andersen and Williams, 1988) through appraisals that lead to individual neurophysiological responses (McEwen, 2007; Mellalieu et al., 2021). Using athlete management systems, practitioners aim to measure the most relevant variables related to training load while keeping the number of questionnaires and the burden for players to a minimum. While monitoring academic stress during specific periods could be a useful possibility, a more global approach to monitoring non-training stressors may be more effective for practitioners than focusing on specific stressors (Mellalieu et al., 2021). Still, given the mixed findings in studies associating pre-training stress levels with training load (Haddad et al., 2013; Long et al., 2025; Nobari et al., 2021), further research remains warranted.

Our final multilevel models showed associations between pre-training academic stress and mental fatigue with RPE; this only partly supported our hypothesis because neither was associated with TRIMP. Both RPE and TRIMP are considered valid measures for internal load in soccer. However, RPE is deemed a subjective measure, whereas TRIMP is a more objective measure. There is evidence that subjective measures have better responsiveness compared to objective measures (Saw et al., 2016). Although it is recognized that stress could increase HR, it did not influence the TRIMP score when corrected for external load (McEwen, 2007). Since TRIMP is calculated using HR values above 65% of a player’s maximum, the high-intensity nature of training sessions likely elevates HR to such an extent that any potential increase due to stress is either masked by the overall intensity or too small to be detected. Moreover, stress seems to cause more attentional and somatic changes, such as distractibility and reduced coordination, which are not directly related to HR (Andersen and Williams, 1988; Soligard et al., 2016). In line with this reasoning, Otter et al. (2016) found that in a group of runners, a negative life event significantly increased general stress, the running economy during a submaximal test was impaired, but the HR response was not affected. This aligns with previous findings that subjective measures possess superior sensitivity to changes in training load compared to objective measures, although a long-term effect of stress on HR cannot be ruled out (Saw et al., 2016). This study adds further evidence that subjective measures, such as RPE, are likely more sensitive than objective measures for detecting moderating effects of non-training stressors.

### 4.2 Limitations

This was the first study to delve into the relationship between academic stress, mental fatigue and training load, combining multiple research domains and thus taking more of the real-world complexity into account that coaches and other practitioners have to deal with. Moreover, the use of real-world data combined with sophisticated statistical analysis to investigate the relationship means that the found associations reflect cases that could occur in daily practice. Nevertheless, this approach also has its limitations. Due to the real-world setting, missing data was prevalent. It remains difficult to keep players fully engaged with the study’s aim and provide information daily when other commitments are also required from the players. Moreover, although missing at random was assumed, missing not at random can never be fully ruled out, suggesting some caution in interpreting the results. Perhaps future research can identify measures that are less invasive for players but that do reliably measure what we intend to measure. These concerns potentially limit the ability to generalize the results. An implication for further research would therefore be to repeat this study in nations with different educational systems or at other clubs with different beliefs towards education. Other limitations include the use of a non-validated single-item measure for academic stress. Despite our efforts, players could have interpreted the question differently than intended. Finally, other factors could have been of interest for this study, offering additional perspectives on the relationships observed in this study. However, with the real-world complexity of training load monitoring in mind, not all potentially important factors can be included, as the burden on players should be kept to a minimum. Operating within these constraints provided by the real-world complexity of training load monitoring, our study adds to the limited research on the relationship between non-training stressors and training load. This approach enhances the ecological validity of our findings while providing new potential avenues for research.

### 4.3 Practical implications

Practitioners face a complicated task managing adolescent players’ day-to-day load and keeping track of influencing factors. They should be aware that non-training stress, such as academic stress, could not only increase the risk of illness and injury (Ivarsson and Johnson, 2010; Hamlin et al., 2019) but also directly influence training load. Besides academic stress, higher levels of mental fatigue were also associated with increased RPE, possibly leading to undesired training load alterations. Therefore, involvement in players’ dual careers seems crucial in understanding and acting proactively on potential non-training stressors. Coaches and supporting staff should adopt a holistic approach by monitoring general non-training stress and mental fatigue. High levels of non-training stress, mental fatigue, or notable within-player deviations should prompt practitioners to adjust training programs accordingly. For soccer players involved in education, however, monitoring academic stress around weeks with expected high academic stress could be an interesting alternative.

## 5 Conclusion

We examined the extent to which academic stress and mental fatigue predict internal load in adolescent soccer players after controlling for external load. We found that academic stress and mental fatigue are positively associated with RPE, but not with TRIMP. Therefore, coaches need to be vigilant when they notice deviations from normal scores or when high levels of academic stress or mental fatigue are present. Coach involvement in players’ dual careers is important, given the potential for non-training stressors. Although educational responsibilities are an important part of many adolescent soccer players’ personal lives, it is likely not desirable for all involved to separately monitor all types of non-training stress. Therefore, adopting a holistic monitoring strategy that integrates global assessments of non-training stress and mental fatigue is recommended. We recommend that future research be conducted to add evidence that non-training stress significantly influences RPE. Finally, RPE should be used to monitor internal load, as our results support superior sensitivity for non-training stressors compared to TRIMP.

## Data Availability

The datasets presented in this study can be found in online repositories. The names of the repository and the link can be found at: DataverseNL https://dataverse.nl/dataset.xhtml?persistentId=doi:10.34894/TMRZCQ.
