# Supplementary material for: Academic stress and mental fatigue predict subjective but not objective internal load in adolescent soccer players—a prospective cohort study
Source: Front Psychol. 2026 Mar 17;17:1770781. doi: 10.3389/fpsyg.2026.1770781 (PMC13035501; doi:10.3389/fpsyg.2026.1770781)
Supplement: Supplementary file 1 [file Table_1.docx]

***Supplementary file***

**1. Sensitivity Analysis**

Table S1-S2 contain the sensitivity analyses for both RPE and TRIMPmod, respectively. For Academic Stress (AS), we conducted a sensitivity analysis where we simulated that AS was either under- (+1) or overestimated (-1). Similarly, for Mental Fatigue (MF), we simulated that MF was under- (+10) or overestimated (-10) during the multiple imputation process. Table S1-S2 contain the estimates produced during the sensitivity analyses. For further information on the code, please see the accompanying codes and raw data in the repository (see online article for exact link).

Table S1: Sensitivity Analysis for RPE regarding four delta adjustments

|  | *Fixed Effects* | | | |
| --- | --- | --- | --- | --- |
|  | AS-1 | AS+1 | MF-10 | MF+10 |
| Intercept | 14.317 (± .532)† | 14.436 (± .560)† | 14.240 (± .571)† | 14.054 (±.589)† |
| Academic stress | .214 (± .088)* | .097 (± .082) | .257 (±. 103)* | .279 (± .116)* |
| Mental Fatigue | .019 (± .006)‡ | .021 (± .006)† | .017 (± .005)‡ | .014 (± .006)* |
| Total distance | .0002 (± .0001)‡ | .0002 (± .0001)‡ | .0003 (± .0001)‡ | .0002 (± .0001)‡ |
| High-speed running | .003 (± .001)† | .003 (± .001)† | .003 (± .001)† | .003 (± .001)† |
| Accelerations | .004 (± .005) | .003 (± .005) | .003 (± .005) | .005 (± .005) |
| Decelerations | .003 (± .005) | .005 (± .006) | .004 (± .006) | .003 (± .005) |
| Team | -.854 (± .316)‡ | -.858 (± .329)‡ | -.875 (±.316)‡ | -.853 (± .325)‡ |
|  |  |  |  |  |
|  | *Random Effects* | | | |
|  | Variance | | | |
| Intercept | .633 | .666 | .634 | .645 |
| Residual | 3.882 | 3.889 | 3.876 | 3.872 |
| AS = Academic Stress; MF = Mental Fatigue  † p < .001; ‡ p < .01; * p < .05  *Note: For total distance, 4 decimal places are used instead of 3 to prevent masking meaningful effects* | | | | |

Table S2: Sensitivity Analysis for TRIMPmod regarding four delta adjustments

|  | *Fixed Effects* | | | |
| --- | --- | --- | --- | --- |
|  | AS-1 | AS+1 | MF-10 | MF+10 |
| Intercept | 156.747 (± 12.421)† | 154.502 (± .13.028)† | 156.747 (± 12.422)† | 154.454 (± 12.942)† |
| Academic stress | 0.105 (± 1.721) | .535 (± 1.671) | .105 (± 1.721) | .311 (± 2.221) |
| Mental Fatigue | .066 (± .099) | .077 (± .110) | .066 (± .099) | .103 (± .094) |
| Total distance | .011 (± .002)† | .010 (± .001)† | .0106 (± .002)† | .010 (± .001)† |
| High-speed running | .019 (± .009)* | .019 (± .009)‡ | .019 (± .009) | .020 (± .009)* |
| Accelerations | .279 (± .083)‡ | .274 (± .109)* | .279 (± .083)‡ | .248 (± .091)‡ |
| Decelerations | .403 (± .091)† | .435(± .109)‡ | .403 (± .091)† | .451 (± .102)† |
| Team | -17.466 (± 7.603)* | -16.895 (± 7.476)* | -17.466 (± 7.603)* | -16.647 (± 7.419)* |
|  |  |  |  |  |
|  | *Random Effects* | | | |
|  | Variance | | | |
| Intercept | 449.608 | 405.721 | 446.274 | 426.655 |
| Residual | 1846.056 | 1227.997 | 1835.366 | 1845.430 |
| AS = Academic Stress; MF = Mental Fatigue  † p < .001; ‡ p < .01; * p < .05 | | | | |
